# Supplementary material for: Multimer Embedding Approach for Molecular Crystals up to Harmonic Vibrational Properties
Source: J Chem Theory Comput. 2023 Dec 18;20(1):357–67. doi: 10.1021/acs.jctc.3c01082 (PMC10782452; doi:10.1021/acs.jctc.3c01082)
Supplement: Supplementary file 1 — ct3c01082_si_001.pdf [file ct3c01082_si_001.pdf]

Supporting Information for:

A Multimer Embedding Approach for Molecular Crystals up to Harmonic Vibrational Properties

Johannes Hoja*[a], Alexander List[a], and A. Daniel Boese*[a]

[a] Department of Chemistry, University of Graz, Heinrichstraße 28/IV, 8010 Graz, Austria

* Correspondence to: johannes.hoja@uni-graz.at, adrian_daniel.boese@uni-graz.at

Contents

1 Number of Dimers and Trimers **2**

2 Lattice Energies **3**

3 Unit-Cell Volumes **6**

4 Phonon Densities of States **8**

1 Number of Dimers and Trimers

Table S1: Number of identified unique dimers and trimers for a given cutoff distance for the X23 set evaluated on top of PBE0+MBD/light optimized structures; Z lists the number of molecules within the unit cell and n is the number of atoms per molecule.

			Dimers						Trimers			
	n	Z	3 Å	4 Å	5 Å	6 Å	7 Å	8 Å	3 Å	4 Å	5 Å	6 Å
1,4-Cyclohexanedione	16	2	6	8	9	14	20	28	8	16	20	51
Acetic acid	8	4	10	16	22	34	52	70	8	32	64	154
Adamantane	26	2	4	4	13	15	18	18	4	4	12	24
Ammonia	4	4	2	3	5	8	13	16	7	11	21	72
Anthracene	24	2	6	7	8	15	18	20	8	12	14	59
Benzene	12	4	6	6	9	14	20	28	8	8	20	51
Carbon dioxide	3	4	0	2	3	5	8	14	0	8	10	27
Cyanamide	5	8	10	15	18	43	56	72	6	28	40	260
Cytosine	13	4	4	7	10	17	21	28	2	12	25	79
Ethyl carbamate	13	2	13	15	19	35	41	53	20	28	46	166
Formamide	6	4	9	14	23	37	59	75	6	24	64	184
Hexamine	22	2	6	6	6	10	19	19	1	1	1	33
Imidazole	9	4	10	15	18	33	46	58	6	28	38	142
Naphthalene	18	2	6	7	8	17	18	21	8	12	14	77
Oxalic acid α	8	4	6	8	12	17	26	34	8	16	36	76
Oxalic acid β	8	2	5	7	10	18	26	32	4	12	24	87
Pyrazine	10	2	4	5	5	11	13	20	4	7	7	39
Pyrazole	9	8	22	26	40	72	94	122	24	40	100	350
s-Triazine[a]	9	6	36	42	60	96	120	174	36	72	144	372
s-Trioxane	12	6	6	6	8	14	16	22	8	8	16	68
Succinic acid	14	2	5	7	11	16	24	31	4	12	28	68
Uracil	12	4	8	16	17	30	40	58	4	32	36	116
Urea	8	2	3	6	6	11	16	19	2	9	9	46

[a] Optimized without symmetry constraints.

2 Lattice Energies

Table S2: Lattice energies in kJ/mol calculated for the respective method with light species default settings on top of PBE0+MBD/light-optimized structures (part 1).

System	PBE0+MBD	PBE+MBD	ME1	ME2(3Å)	ME2(4Å)	ME2(5Å)	ME2(6Å)
1,4-Cyclohexanedione	-99.6	-92.7	-95.0	-96.5	-95.6	-95.6	-95.7
Acetic acid	-80.1	-77.3	-78.0	-77.6	-77.4	-77.5	-77.6
Adamantane	-81.3	-76.7	-79.6	-77.6	-77.6	-77.4	-77.4
Ammonia	-42.7	-45.3	-45.2	-39.7	-39.2	-39.2	-38.8
Anthracene	-124.5	-111.1	-114.8	-121.5	-121.4	-121.4	-121.6
Benzene	-60.4	-56.2	-57.4	-57.8	-57.8	-57.8	-57.8
Carbon dioxide	-28.0	-24.6	-27.7	-27.7	-26.3	-26.3	-26.3
Cyanamide	-90.5	-92.1	-92.9	-87.8	-87.0	-86.9	-86.7
Cytosine	-171.5	-164.5	-164.6	-165.7	-167.3	-167.8	-168.0
Ethyl carbamate	-95.1	-91.1	-93.4	-92.0	-91.7	-91.3	-91.2
Formamide	-85.9	-84.8	-83.6	-83.8	-83.0	-82.3	-81.9
Hexamine	-95.9	-89.1	-91.0	-94.0	-94.0	-94.0	-93.8
Imidazole	-98.3	-96.1	-96.5	-95.7	-95.9	-95.8	-95.4
Naphthalene	-91.6	-82.8	-85.2	-88.7	-88.5	-88.5	-88.7
Oxalic acid α	-107.4	-98.1	-102.7	-103.9	-104.0	-103.9	-103.9
Oxalic acid β	-107.6	-100.3	-103.4	-102.8	-102.5	-102.5	-102.5
Pyrazine	-69.5	-65.8	-67.4	-65.1	-66.5	-66.5	-66.4
Pyrazole	-87.5	-85.0	-85.6	-85.1	-84.9	-84.9	-84.8
s-Triazine	-64.0	-58.7	-60.7	-59.0	-60.4	-60.2	-60.3
s-Trioxane	-70.6	-63.7	-65.8	-66.1	-66.1	-66.1	-66.0
Succinic acid	-142.2	-136.5	-137.2	-138.0	-136.5	-136.6	-136.6
Uracil	-147.2	-139.8	-140.3	-142.3	-143.3	-143.3	-143.5
Urea	-112.5	-109.8	-110.1	-110.6	-109.0	-109.0	-107.8

Table S3: Lattice energies in kJ/mol calculated for the respective method with light species default settings on top of PBE0+MBD/light-optimized structures (part 2).

System	ME2(7Å)	ME2(8Å)	ME3(3Å)	ME3(4Å)	ME3(5Å)	ME3(6Å)
1,4-Cyclohexanedione	-95.7	-95.7	-99.6	-100.0	-100.0	-100.2
Acetic acid	-77.6	-77.5	-78.5	-80.6	-81.1	-81.1
Adamantane	-77.4	-77.4	-81.5	-81.5	-81.8	-81.8
Ammonia	-38.9	-38.9	-42.8	-42.9	-43.1	-43.9
Anthracene	-121.6	-121.6	-124.2	-124.3	-124.4	-125.0
Benzene	-57.8	-57.8	-60.2	-60.2	-60.7	-61.0
Carbon dioxide	-26.3	-26.2	-27.7	-28.2	-28.6	-28.5
Cyanamide	-86.7	-86.6	-90.0	-91.5	-91.9	-91.5
Cytosine	-167.5	-166.8	-167.2	-171.7	-172.4	-173.5
Ethyl carbamate	-91.2	-91.3	-95.3	-95.6	-95.7	-95.9
Formamide	-81.9	-82.0	-85.3	-87.0	-87.4	-87.6
Hexamine	-93.8	-93.8	-96.0	-96.0	-96.0	-96.2
Imidazole	-95.4	-95.4	-96.8	-99.0	-99.0	-99.1
Naphthalene	-88.6	-88.6	-91.4	-91.5	-91.6	-92.2
Oxalic acid α	-103.9	-103.9	-107.3	-107.2	-107.9	-108.0
Oxalic acid β	-102.5	-102.5	-105.8	-107.5	-108.6	-109.1
Pyrazine	-66.3	-66.4	-66.7	-69.9	-69.9	-70.3
Pyrazole	-84.9	-84.9	-87.0	-87.4	-87.8	-87.9
s-Triazine	-60.3	-60.2	-61.1	-64.5	-64.7	-64.7
s-Trioxane	-66.3	-65.9	-69.4	-69.4	-70.0	-70.0
Succinic acid	-136.8	-136.7	-140.6	-142.9	-143.6	-143.5
Uracil	-143.4	-143.5	-143.9	-147.8	-147.8	-148.2
Urea	-108.9	-109.1	-113.0	-113.1	-113.1	-112.4

Table S4: Lattice energies in kJ/mol calculated for the respective method with tight settings on top of PBE0+MBD/light-optimized structures.

System	PBE0+MBD	PBE+MBD	ME1	ME2(3Å)	ME2(4Å)	ME3(3Å)	ME3(4Å)
1,4-Cyclohexanedione	-93.4	-89.6	-91.3	-90.2	-89.2	-93.5	-93.8
Acetic acid	-76.4	-74.8	-74.9	-74.4	-73.8	-75.5	-77.0
Adamantane	-79.3	-77.7	-80.6	-75.2	-75.2	-79.4	-79.4
Ammonia	-40.8	-42.5	-42.3	-37.9	-37.3	-41.2	-41.8
Anthracene	-110.8	-103.8	-107.0	-107.1	-106.9	-110.9	-111.1
Benzene	-55.4	-53.3	-54.4	-52.4	-52.4	-55.4	-55.4
Carbon dioxide	-24.5	-21.8	-23.6	-23.6	-22.7	-23.6	-24.6
Cyanamide	-89.6	-91.3	-91.2	-87.5	-86.4	-89.1	-90.5
Cytosine	-162.4	-158.5	-157.7	-159.5	-158.5	-160.6	-163.0
Ethyl carbamate	-90.8	-88.9	-90.3	-88.0	-87.7	-91.3	-91.5
Formamide	-82.2	-81.4	-79.5	-81.2	-79.8	-82.3	-83.7
Hexamine	-89.5	-85.9	-88.3	-85.1	-85.1	-89.8	-89.8
Imidazole	-92.3	-91.8	-91.7	-90.4	-89.9	-91.4	-93.1
Naphthalene	-82.7	-77.9	-80.1	-79.2	-79.0	-82.7	-82.9
Oxalic acid α	-98.5	-91.7	-94.1	-95.5	-95.5	-98.5	-98.3
Oxalic acid β	-98.7	-93.8	-94.9	-95.3	-94.5	-97.6	-98.9
Pyrazine	-61.9	-60.5	-61.6	-58.4	-58.4	-60.3	-62.6
Pyrazole	-81.5	-80.7	-80.6	-79.2	-78.9	-81.3	-81.6
s-Triazine	-57.5	-55.0	-56.3	-54.0	-54.1	-56.1	-58.4
s-Trioxane	-63.1	-59.8	-61.0	-58.4	-58.4	-62.3	-62.3
Succinic acid	-135.5	-131.5	-131.4	-131.7	-130.2	-134.3	-136.0
Uracil	-138.9	-134.4	-134.1	-136.8	-135.5	-137.8	-139.6
Urea	-110.8	-108.2	-107.8	-109.9	-108.2	-111.7	-111.9

Table S5: Errors of the calculated lattice energies of the X23 set compared to PBE0+MBD. All energies were calculated using tight settings on top of the PBE0+MBD/light-optimized structures. The mean error (ME), the mean absolute error (MAE), and the maximal error (MAX) are given in kJ/mol while the mean relative error (MRE), the mean absolute relative error (MARE), and the maximal relative error (RMAX) are given in %.

Method	ME	MAE	MAX	MRE	MARE	RMAX
PBE+MBD	2.7	3.0	7.0	-3.1	3.7	11.0
ME1	1.8	2.2	4.8	-1.8	2.4	4.5
ME2(3Å)	2.8	2.8	4.7	-3.6	3.6	7.4
ME2(4Å)	3.4	3.4	5.3	-4.4	4.4	8.6
ME3(3Å)	0.4	0.7	1.8	-0.6	0.9	3.5
ME3(4Å)	-0.5	0.5	1.4	0.6	0.7	2.4

3 Unit-Cell Volumes

Table S6: Unit-cell volumes in $Å^3$ calculated for the respective method with light species default settings.

System	PBE0+MBD	PBE+MBD	ME1	ME2(3Å)	ME2(4Å)	ME2(5Å)
1,4-Cyclohexanedione	271.3	280.7	278.3	274.7	275.8	275.8
Acetic acid	291.5	300.0	299.5	295.4	295.6	295.6
Adamantane	369.2	383.4	377.9	375.7	376.7	376.8
Ammonia	123.9	124.8	124.0	130.1	130.6	131.9
Anthracene	444.0	460.1	454.9	448.2	448.1	448.2
Benzene	453.2	469.0	464.1	461.0	461.0	461.0
Carbon dioxide	179.4	188.2	185.6	185.5	184.3	184.4
Cyanamide	411.4	422.4	417.1	422.8	430.2	428.8
Cytosine	457.6	478.8	474.3	475.5	466.1	465.6
Ethyl carbamate	237.2	245.1	242.2	240.5	240.4	240.9
Formamide	218.1	224.7	224.6	224.8	224.5	225.5
Hexamine	314.6	330.1	324.6	321.9	321.9	321.9
Imidazole	344.7	358.3	355.2	354.9	353.3	353.7
Naphthalene	333.7	346.1	342.1	337.8	337.8	337.9
Oxalic acid α	303.3	315.9	314.5	307.1	307.1	307.1
Oxalic acid β	152.5	159.4	158.6	155.7	155.9	155.9
Pyrazine	191.9	201.0	199.2	200.2	198.7	198.7
Pyrazole	689.8	717.8	711.3	701.8	702.1	702.3
s-Triazine	549.1	567.2	563.4	562.8	558.7	559.6
s-Trioxane	575.2	611.5	606.7	600.3	600.3	600.4
Succinic acid	237.3	246.4	244.3	241.4	241.8	241.9
Uracil	445.8	462.7	459.1	455.2	450.7	451.0
Urea	140.5	144.1	143.7	142.8	143.4	143.4

Table S7: Unit-cell volumes in Å^3 calculated for the respective method with tight settings.

System	PBE0+MBD	PBE+MBD	ME1	ME2(3Å)	ME2(4Å)
Acetic acid	292.4	298.5	298.0	296.3	296.9
Ammonia	124.1	125.2	125.0	128.5	129.1
Carbon dioxide	182.6	190.7	188.5	188.5	186.6
Ethyl carbamate	237.2	242.2	240.2	239.7	240.2
Oxalic acid β	154.1	159.6	158.6	156.2	156.9
Pyrazine	197.6	201.6	200.0	201.2	200.6
Succinic acid	238.7	245.4	243.8	241.9	242.3
Urea	141.9	144.2	143.7	142.3	143.0

Table S8: Errors of calculated cell volumes (in %) of a reduced set containing 8 systems from X23 calculated with tight settings compared to PBE0+MBD results.

Method	MRE	MARE	RMAX
PBE+MBD	2.5	2.5	4.4
ME1	1.8	1.8	3.2
ME2(3Å)	1.8	1.8	3.5
ME2(4Å)	1.8	1.8	4.1

4 Phonon Densities of States

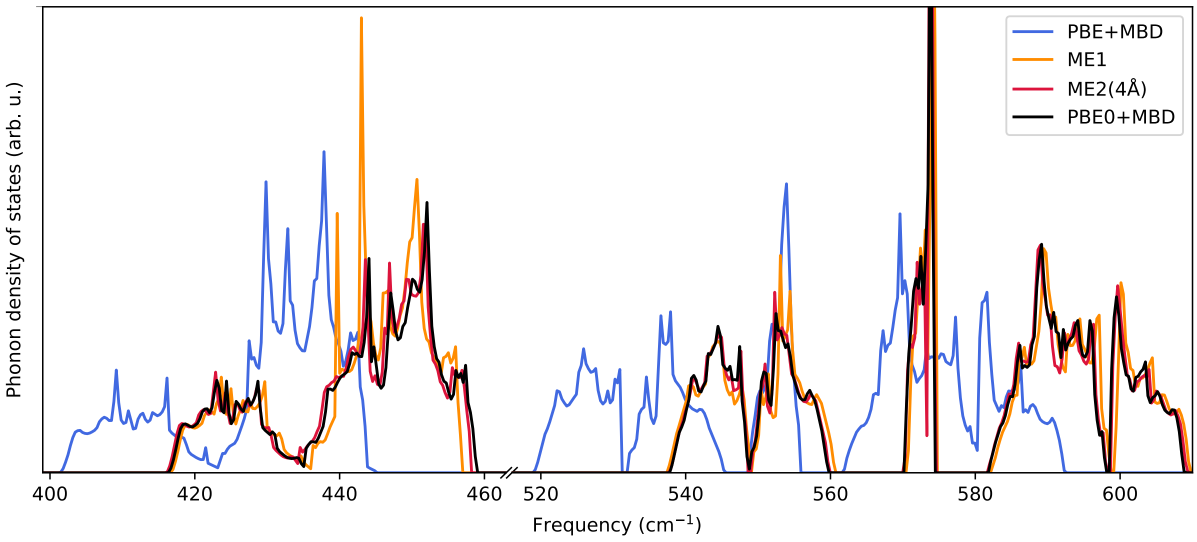

Figure S1: Phonon density of states of uracil between 400 and 610 cm^{-1} calculated on top of internally relaxed structures using the PBE0+MBD/light lattice vectors for several methods.

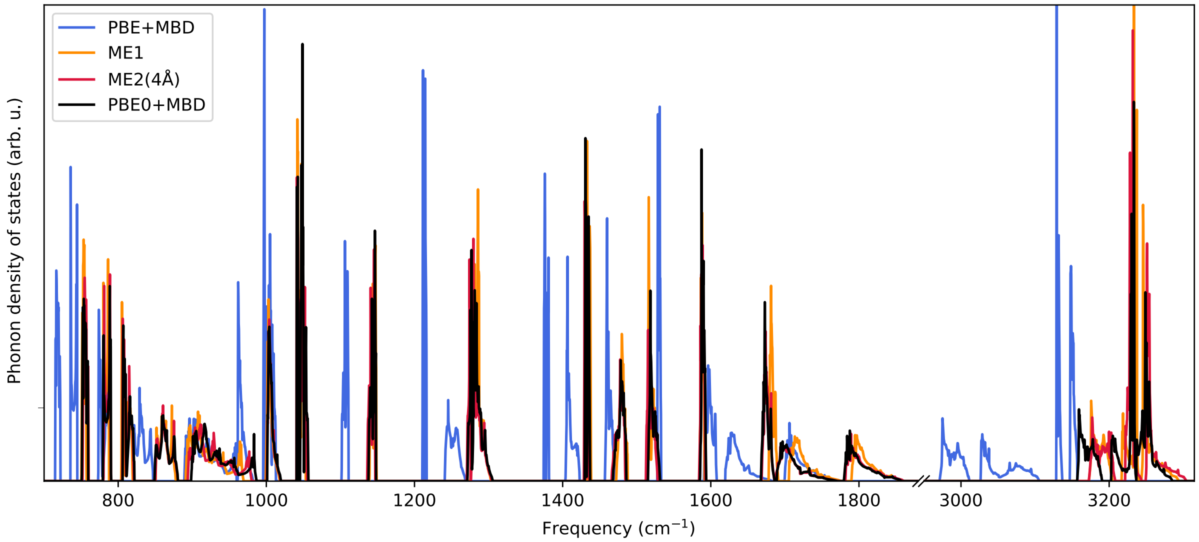

Figure S2: Phonon density of states of uracil above 700 cm^{-1} calculated on top of optimized structures for several methods.

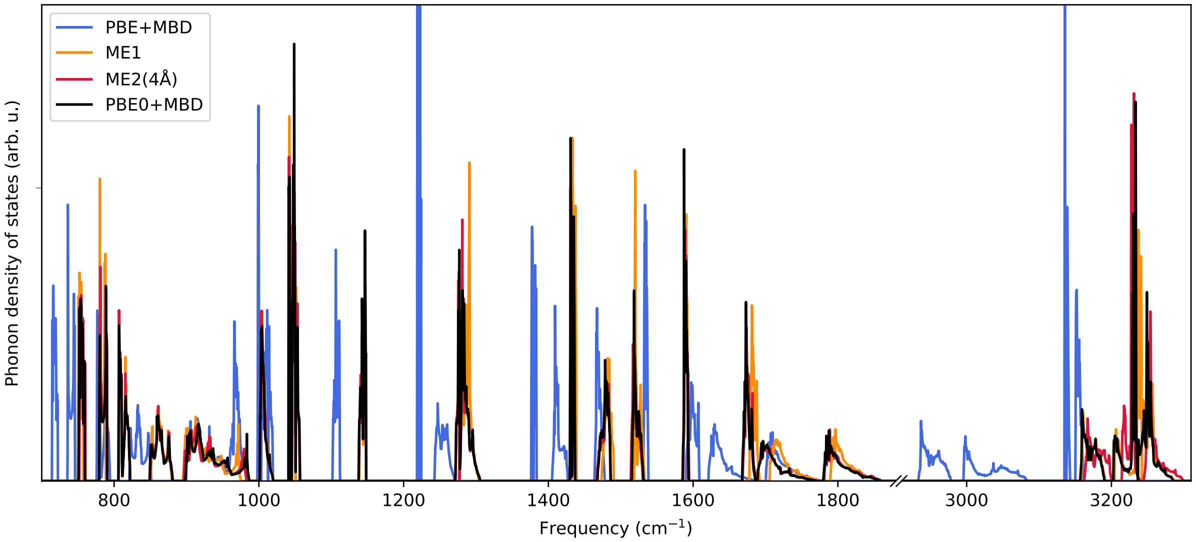

Figure S3: Phonon density of states of uracil above 700 cm^{-1} calculated on top of internally relaxed structures using the PBE0+MBD/light lattice vectors for several methods.

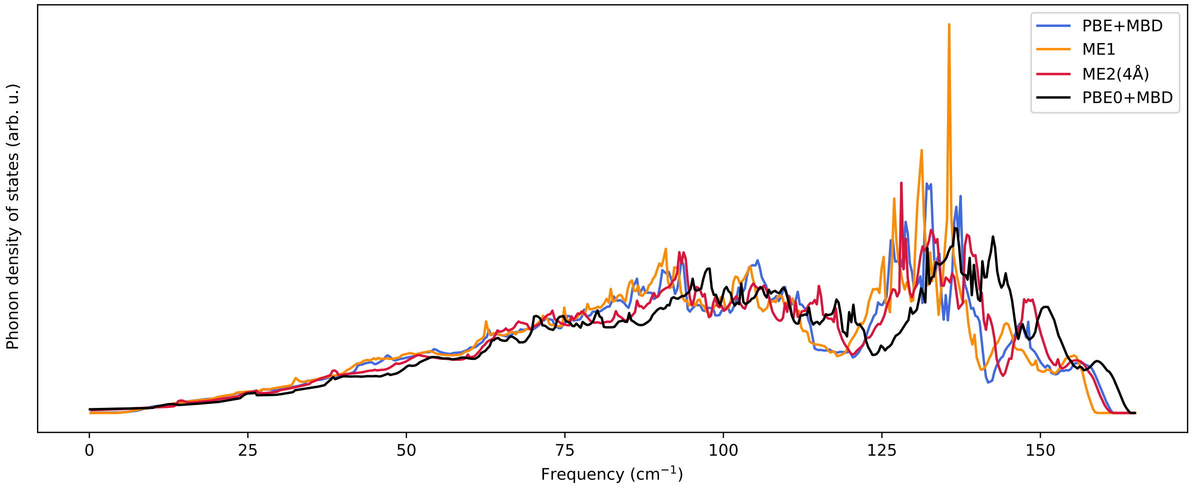

Figure S4: Low-frequency phonon density of states of uracil calculated on top of optimized structures for several methods.

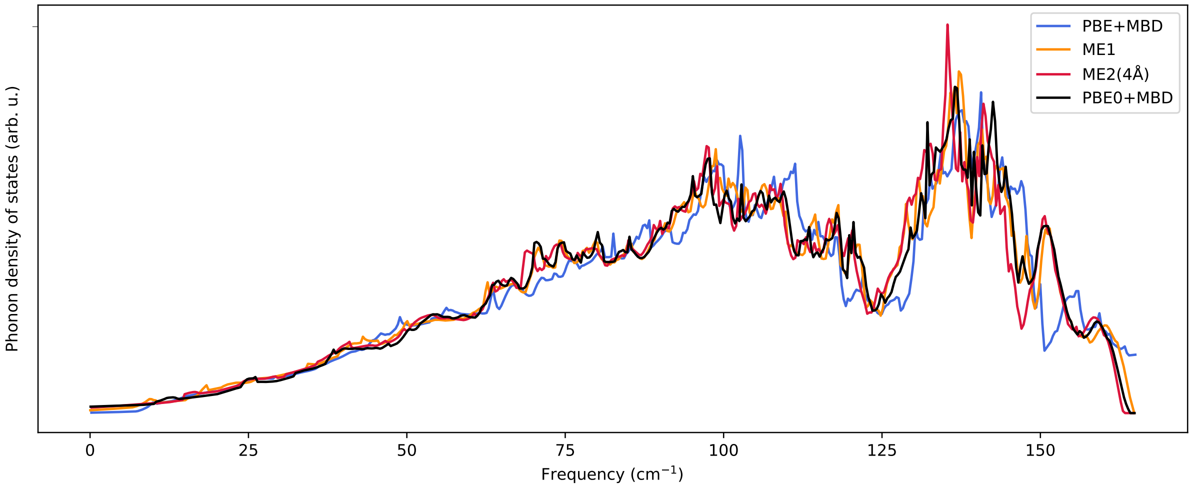

Figure S5: Low-frequency phonon density of states of uracil calculated on top of internally relaxed structures using the PBE0+MBD/light lattice vectors for several methods.
